# Supplementary material for: UVRAG Promotes Tumor Progression through Regulating SP1 in Colorectal Cancer
Source: Cancers (Basel). 2023 Apr 27;15(9):2502. doi: 10.3390/cancers15092502 (PMC10177159; doi:10.3390/cancers15092502)
Supplement: Supplementary file 1 [file cancers-15-02502-s001.zip › cancers-2323172-supplementary.pdf]

Supplementary materials

Table S1. Characteristics of Cohort 1.

Clinical features	Cohort 1
Gender	
Male	161
Female	133
Age	
Mean± SD	59.55 ± 0.7194
T stage	
T1	2
T2	27
T3	213
T4	52
N stage	
N0	111
N1	95
N2	88
M stage	
M0	134
M1	160
TNM stage	
I	22
Π	58
III	54
IV	160
Differentiation	
Mucinous adenocarcinoma	13
Poor	57
Moderate	158
Well	66
Adjuvant chemotherapy	
Yes	228
No	63
Missing data	3
Location	
Colon	177
Rectum	117

Table S2. Characteristics of Cohort 2.

Clinical features	Cohort 2
Gender	
Male	181
Female	115
Age	
Mean± SD	57.92 ± 11.59
Pathologic T stage	
ypT0	11
ypT1-2	96
ypT3-4	189
Pathologic N stage	
ypN0	169
ypN1	81
ypN2	46
ypTNM stage	
ypCR	10
I	78
П	81
III	127
Tumor regression grade(TRG)	
Grade 0-1	95
Grade 2	77
Grade 3	40
Grade 4	10
Differentiation	
ypCR	10
G1-2	206
G3-4	80

Table S3. Characteristics of Cohort 3.

Clinical features	Cohort3
Gender	
Male	33
Female	27
Age	
Mean± SD	56.23±11.82
Pretreatment T stage	
T1-2	7
T3-4	53
Pretreatment N stage	
N0	6
N1-2	54
Post-treatment T stage	
T0	5
T1-2	18
T3-4	37
Post-treatment N stage	
N0	41
N1-2	19
Venous invasion	
Positive	5
Negative	55
Tumor regression grade(TRG)	
Grade 0-1	19
Grade 2-3	30
Grade 4	11

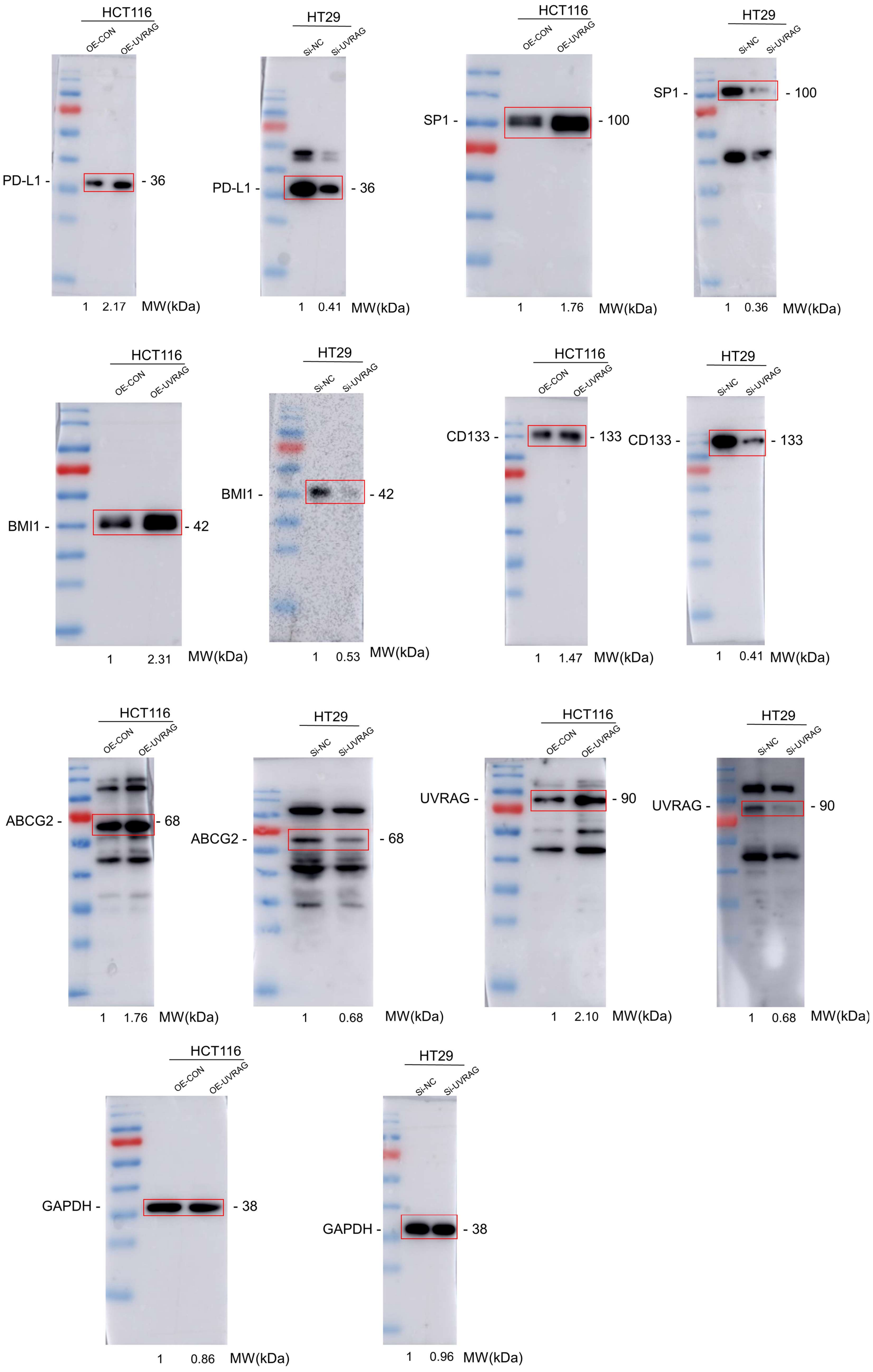

Figure S1. Original, uncropped Western Blot images.
